# E-cigarettes and smoking cessation among adolescent smokers

**DOI:** 10.1038/s41598-022-22344-4

**Published:** 2022-11-14

**Authors:** Li-Yin Lin, Yu-Ning Chien, Yi-Hua Chen, Russell Shean, Chi-Yi Wu, Shih-Chang Huang, Hung-Yi Chiou

**Affiliations:** 1 Department of Leisure Industry and Health Promotion, National Taipei University of Nursing and Health Sciences, 365 MingDe Road, Beitou District, Taipei, 11219 Taiwan; 2 Department of Health and Welfare, University of Taipei, Taipei, Taiwan; 3 School of Public Health, College of Public Health, Taipei Medical University, 250 Wuxing St, Xinyi Dist., Taipei City, 110 Taiwan; 4 Master in Global Health and Development Program, College of Public Health, Taipei Medical University, 250 Wuxing St., Taipei City, 110 Taiwan; 5 Institute of Population Health Sciences, National Health Research Institutes, Zhunan Town, Miaoli County, 35053 Taiwan; 6 Chung-Hua Institution for Economic Research, No.75, Changxing St., Da-an Dist., Taipei City, 106 Taiwan; 7 School of Public Health, College of Public Health, Taipei Medical University, 250 Wuxing St, Taipei City, 110 Taiwan

**Keywords:** Health care, Risk factors

## Abstract

Smokers of any age can reap substantial health benefits from quitting or reducing their smoking. E-cigarettes have been promoted as a potentially promising product for tobacco harm reduction because e-cigarettes deliver nicotine vapor without many of the hazardous chemical combustion byproducts produced by combustible cigarette smoking. However, there remains an ongoing debate on whether the use of e-cigarettes is effective in combustible cigarette smoking cessation or reduction in both adolescents and adults. Our study uses data from the 2015 (baseline) and from the 2017 (follow-up) waves of the Taiwan Adolescent to Adult Longitudinal Study (TAALS), which is a large nationwide representative cohort study of health behaviors among adolescents in Taiwan. We analyzed the data using logistic regression and multivariate regression with a post-stratification weighting procedure. Among the 474 adolescent combustible cigarette users at baseline, the use of e-cigarettes had no association with smoking cessation (aRR = 0.99, 95% CI = 0.66, 1.50). Furthermore, the use of e-cigarettes was also not associated with change in combustible cigarette consumption among all adolescent combustible cigarette users at follow-up (Coef. = 0.62, 95% CI =  − 36.85, 38.09). In summary, our findings suggest that e-cigarettes may not aid tobacco control among adolescent smokers. Policy makers should be cautious of the potential harms that e-cigarette may bring to young people when they are developing e-cigarette regulations.

## Introduction

E-cigarettes have been the most commonly used tobacco product among adolescents in the United States since 2014^1,2^. After increasing between 2017 and 2019, use of e-cigarettes went down among middle and high school students in the United States from 2019 to 2020^3–5^. However, in 2020, 19.6% of high school students in the United States , still reported that they used e-cigarettes in the past 30 days^6^. Epidemiological studies in Asia have suggested a relatively low prevalence of e-cigarette use: < 1% in Indonesia and Malaysia in 2011^7^, and 2.3% in Hong Kong in 2014^8^. In Taiwan, the prevalence of e-cigarette use among adolescents (ages 12–17) is around 0.8% according to the 2014 National Survey of Substance Use in Taiwan^9^. A different study, the Taiwan Global Youth Tobacco Survey (TGYTS) estimated a higher prevalence. TGYTS found that prevalence of current use of e-cigarettes among adolescents (ages 12–18) in Taiwan was on average 3.1% between 2014 and 2016, with 52% being dual users of combustible cigarettes and e-cigarettes^10^. Electronic cigarettes (e-cigarettes) have gained the attention of smokers due to their ability to closely simulate the behavioral experience of smoking, as well as for their ability to deliver a dose of nicotine without involving the combustion of tobacco.

### Why e-cigarettes cause health concerns

For adolescents, e-cigarettes may be an attractive alternative to combustible cigarettes because e-cigarettes are perceived as less harmful and are available in many different flavors^11^. Most e-cigarettes work in a similar way. Puffing activates a battery-powered heating device. This heats up the liquid in a cartridge, turning it into vapors that are inhaled^12,13^. Vaping exposes the lungs to a variety of chemicals. These may include the main active chemicals in tobacco (nicotine) or marijuana (THC), added flavors, and other ingredients that are added to vaping liquids. Also, other chemicals can be produced during the vaporization process^14,15^. One harmful chemical that has been found in e-cigarettes is called vitamin E acetate, which is sometimes used as an additive in THC-containing vape products. The Center of Disease Control (CDC) has identified it as a “chemical of concern” among people with vaping-associated lung injuries^16^. A 2014 American Association policy statement reviewed e-cigarette safety and concluded that a variety of evidence suggests that e-cigarettes are likely significantly less toxic than traditional cigarettes, with the caveats that more evidence is needed and that certain components of e-cigarettes, particularly some flavorings and propylene glycol, may be toxic when inhaled^17^.

### Issues related to e-cigarettes use and combustible tobacco control

In our previous study, we found that e-cigarette use was associated with an increased odds of smoking initiation among adolescents in Taiwan^18^. The European Tobacco Products Directive has stated: “Electronic cigarettes can develop into a gateway to nicotine addiction and ultimately traditional tobacco consumption, as they mimic and normalize the action of smoking”^12^, while the Australian National Health and Medical Research Council concluded that actions should be taken to minimize the harm of e-cigarettes to users and to protect vulnerable groups such as youth^13^.

On the other hand, e-cigarettes may have potential roles to play in both combustible cigarette smoking cessation and tobacco harm reduction^13,19^. A major UK clinical trial published in 2019 found that, when combined with face-to-face counseling support, people who used e-cigarettes to quit combustible cigarette smoking were twice as likely to succeed as people who used other nicotine replacement products, such as gum or patches^20^. In a nationally representative US Population Assessment of Tobacco and Health (PATH) Study, that 3093 quit attempters, found using Electronic Nicotine Delivery System (ENDS) to quit cigarettes increased the probability of persistent cigarette abstinence (≧ 30 days)^21^.

Although some studies suggest that the use of e-cigarette as a smoking cessation aid appear to be useful^22^, e-cigarettes may raise a public health concern if their use leads to increases in youth combustible smoking initiation or consumption in excess of what would have happened in the absence of e-cigarettes^23,24^. In a literature review on the e-cigarette phenomenon, researchers found there was a need for further research to answer key questions about the safety, patterns of use, effectiveness for combustible tobacco smoking cessation and regulatory issues associated with the use of e-cigarettes, especially as it relates to adolescents^12^. The aim of our study is to investigate the association between e-cigarette use and combustible tobacco smoking cessation or reduction. We have proposed two hypotheses for our study. Hypothesis #1: Use of e-cigarettes at baseline are not effective in smoking cessation in adolescent population. The pooled results from nine cohort studies suggested that e-cigarettes were not associated with smoking cessation^25^. One systematic review reported that the odds of cessation were 28% lower in the e-cigarette group than the non-use group (OR = 0.72; 95% CI 0.57–0.91; 20 trials, n = 35,5011)^2^. Moreover, the intension of using e-cigarette to quit smoking is another key determinant. Studies have shown that e-cigarettes are useful tools for adults who are motivated to quit^26^. By contrast, according to the Taiwan Global Youth Tobacco Survey (GYTS) findings, adolescents are using e-cigarettes because they think e-cigarettes are less harmful than combustible cigarettes, having more unique flavors, and it looks fashionable to smoke e-cigarettes^27^. Therefore, we postulate that due to demographic differences, use of e-cigarettes may represent as either a quitting aid or possible gateway drug. Hypothesis#2: The use of e-cigarettes may not decrease the consumption of combustible cigarettes in adolescent population. There is quite some extensive evidence showing a positive association between e-cigarette use is associated with subsequent cigarette use among adolescent and young adult non-smokers^28,29^. However, one issue remains uncertain is whether e-cigarettes will increase combustible cigarette consumption among adolescent population. Although many smokers use e-cigarettes to quit smoking, most continue to smoke while vaping. This dual use might delay smoking cessation and increase toxicant exposure. Therefore, our study aims to investigate whether the use of e-cigarettes increases consumption of combustible cigarettes, particularly in this unique adolescent population.

## Materials and methods

### Data source

**Figure 1:**
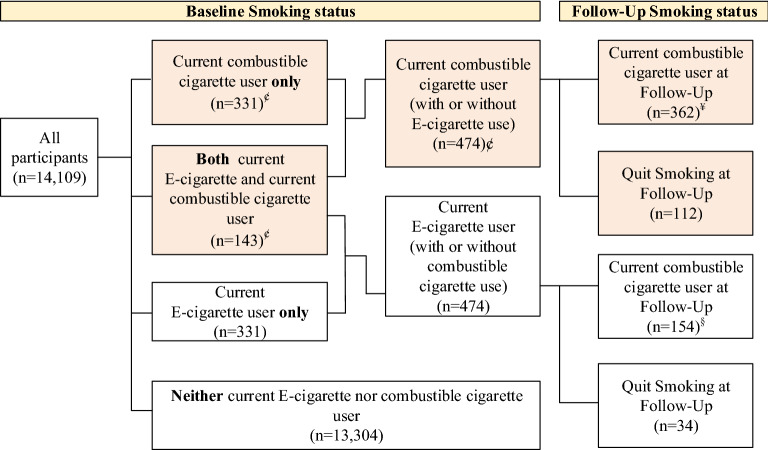
Participants stratified by smoking status – Unweighted data (N = 14,109). *Note*: ¢ is the sample size which is used to analyze the association between use of e-cigarettes at baseline and smoking cessation in adolescent smokers. ¥ is the sample size which is used to analyze the change in total monthly combustible cigarette consumption from baseline to follow-up. The parts highlighted in light orange are our chosen sample for analysis.

Our study uses data from the Taiwan Adolescent to Adult Longitudinal Study (TAALS), a large nationally representative long-term longitudinal cohort study of health behaviors among adolescents in Taiwan that was conducted between 2015 and 2017^28^. At baseline, the TAALS survey interviewed students in the first year of middle school and students in the first year of high school. Since our participants are minors (age less than 16 years), informed consent form was collected from both a parent/or legal guardian and the participant for study participation. During the first wave of the formal cohort study, 6903 middle school students and 11,742 high school students were interviewed, for a total of 18,645 students. During the second wave, the same cohort of students was re-interviewed in their third year of middle school and third year of high school. A total of 16,265 students were interviewed (6381 middle school students and 9884 high school students), representing a follow-up completion rate of 87.21%. After excluding participants with incomplete records, a final analysis was conducted on 14,109 students. Among those 14,109 students, 474 students were current smokers at the baseline, of whom 331 students used only combustible cigarettes, while the remaining 143 students used both combustible cigarettes and e-cigarettes. Among the 474 students who were current smokers, 362 students were still current smokers and the remaining 112 students had quit smoking at follow-up (Fig. 1). A more complete description of the TAALS Cohort and methodology is described in one of our previous publications^28^. This study was approved by the Joint Institutional Review Board of Taipei Medical University, Taiwan (TMU-JIRB-201410043).

### Measures

#### Definitions of Smoking cessation and changes in total monthly combustible tobacco consumption

Two main variables were being examined: smoking cessation and total monthly combustible tobacco consumption. The following methods were used to measure smoking cessation. First, participants were being asked “Have you ever smoked a cigarette?” and if they answered yes, they were being asked “In the last 30 days how many days have you smoked cigarettes?”. Participants who had smoked any number of cigarettes in the last 30 days were defined as current smokers. At follow-up, participants were identified as either current smoker or quit smoking*.* Smoking cessation was defined as participants who indicated that they were current smokers at baseline, but then indicated that they hadn’t smoked any cigarettes in the last 30 days at follow-up.

The following self-reported questions were used to measure total monthly combustible tobacco consumption: (1) “In the last 30 days, how many days did you smoke cigarettes?”, and (2) “In the last 30 days, on the days you smoked cigarettes, on average how many cigarettes did you smoke each day?”. From these questions, we measured the number of smoking days per month and average number of cigarettes smoked per smoking day. We multiplied the average number of cigarettes smoked per smoking day by the number of smoking days per month to calculate the total monthly combustible tobacco consumption. Next we subtracted the total number of cigarettes smoked each month at follow-up from the number of cigarettes smoked each month at baseline to calculate the change in total monthly combustible cigarette consumption.

#### Independent variables at baseline: current e-cigarette use and current use of other tobacco products

In this study, we asked participants at baseline if they are current users of e-cigarettes or other tobacco products. We measured current e-cigarette use by asking: “In the last 30 days how many days have you used e-cigarettes?” and participants responding between 1 and 30 days, were classified as current e-cigarette users. We measured current other tobacco products use by asking “In the past 30 days have you used other tobacco products (such as: cigars, cigarillos, pipes or water pipes)?”.

#### Demographic characteristics variables

This study also recorded the following demographic characteristic variables for all participants: sex, depression, peer support, age, father’s highest education level (junior high or below, high school, university or higher), mother’s ethnicity (Han Chinese, Aboriginal Taiwanese, or New Immigrant), father’s occupation status (full-time, part-time, unemployed), and family living arrangement status (living with both mother and father, living with both parents and grandparents, living with a single parent, living with only grandparents, and living with someone other than direct kin).

#### Measurements of depressive symptoms and Peer Support Score

Center for Epidemiological Studies Depression Scale (CES-D) is a brief self-report questionnaire used to measure the severity of depressive symptoms^30^. The original CES-D consists of 20 questions that ask about various symptoms of depression (i.e. poor appetite, restless sleep, and feeling lonely) as they have occurred in the past week. Response options range from 0 to 3 for each item (0 = Rarely or None of the Time, 1 = Some or Little of the Time, 2 = Moderately or Much of the time, 3 = Most or Almost All the Time). Scores range from 0 to 60, with high scores indicating greater depressive symptoms^31^.

The level of peer support was quantified using a 5-item questionnaire developed by the U.S. Centers for Disease Control and Prevention (CDC)^32^. Questions were being asked to evaluate the level of peer support received by the participants during the past 6 months. Questions include: “My classmates/friends truly care about things that happened to me”, “when I am in need for help, my classmates/friends will help me”, “I have classmates/friends that I can trust”, “My classmates/friends care about my feelings”, “My classmates/friends only care about themselves”, and “My classmates/friends think that I’m not good enough”. Responses were rated on a 4-point scale ranging from 0 (none of them) to 3 (All of them) and the scores were summed up to yield a total score. The total score ranges from 0 to 15, where a higher score indicates stronger peer support.

### Statistical analysis

The logistic regression was used to evaluate the association between the use of e-cigarettes and future smoking cessation among adolescents. To ensure our analytic results remain nationally representative of adolescents in Taiwan, we conduct weighted adjustment. We divided our sample into 24 subgroups (including 4 geographic areas (North, Center, South, East of Taiwan) × 3 school size (small, medium, large) × gender (male and female)). For each group, we calculated a specific weight as shown in the following equation:

$${W}_{g,s,x}=\frac{\left(\frac{{N}_{g,s,x}}{N}\right)}{\left(\frac{{n}_{g,s,x}}{n}\right)}$$

where g, s, x denotes the geographic area, school of size, and gender. ${n}_{g,s,x}$ denotes the number of individuals in the sample of gender × attending the school of size s in the gth geographic area. N refers to the number of entire population. ${N}_{g,s,x}$ refers to the number of individuals in the population of gender x attending the school of size s in the gth geographic area. For a detailed description on how weighted adjustment was performed, please refer to Chein et al.^28^. We used risk ratio (RR) and risk difference (RD) instead of odds ratio (OR) because the use of RR and RD can better reflect the relative and absolute probability change in smoking cessation in the presence of e-cigarettes. Here the adjusted risk ratio (aRR) and adjust risk difference (aRD) were performed using the “adjrr” command in STATA statistical software (version 16 MP, StataCorp LLC)^33^. *P*-value of < 0.05 was considered significant.

### Informed consent

Informed consent was obtained from all subjects involved in the study.

### Institutional review board statement

The study was conducted according to the guidelines of the Declaration of Helsinki and approved by the Taipei Medical University Joint Institutional Review Board (TMU JIRB), No. 201410043.

## Results

### Characteristics of study participants

In Table 1, the majority of participants among current e-cigarettes users at baseline had never used other tobacco products (63.6%), were senior high school students (81.8%), were male (76.9%), had their father’s highest education level above senior high school, had their mother’s ethnicity being Han Chinese (81.8%), had their parents working full-time (87.4%), and lived with parents or extended family (64.3%). The majority of participants among non-current e-cigarette users at baseline had never used other tobacco products (90.9%), were male (77.0%), were senior high school students (85.2%), had their father’s highest education level above senior high school (50.5%), had their mother’s ethnicity being Han Chinese (81.6%), had their parents working full-time (91.5%), and lived with parents or extended family (63.4%). In addition, participants from the current e-cigarettes users at baseline group had a mean CES-D scale of 8.8 ± 2.9, a mean peer support score of 13.2 ± 2.6, and a mean age of 17.8 ± 1.3. Participants from the non-current e-cigarette users at baseline group had a mean CES-D scale of 8.4 ± 2.8, a mean peer support score of 13.6 ± 2.4, and a mean age of 18.0 ± 1.3.

**Table 1:** Demographic characteristics of current smokers stratified by current E-cigarette use—unweighted versus weighted observations (n = 474).

	Overall case (n = 474)—unweighted	Current smokers at baseline (*n* = 474)				Current smokers at baseline (n = 474)				*P* value
		Current E-cigarettes users at baseline (n = 143)		Not current E-cigarettes users at baseline (n = 331)		Current E-cigarettes users at baseline (n = 143)		Not current E-cigarettes users at baseline (n = 331)		
		Unweighted observation (n)	Unweighted percentage (%)	Unweighted observation (n)	Unweighted percentage (%)	Weighted OBSERVATION (n)	Weighted percentage (%)	Weighted observation (n)	Weighted percentage (%)	
**Quit smoking at follow-up**										
Yes	112	34	23.8	78	23.6	35	24.6	81	24.4	0.982
No	362	109	76.2	253	76.4	107	75.4	249	75.6	
**Ever used other tobacco products (e.g. cigars, water pipe, pipe, cigarillos ) at baseline**										
Yes	*82*	52	36.4	30	9.1	51	36.4	28	8.6	< 0.001***
No	*392*	91	63.6	301	90.9	90	63.6	301	91.4	
**Grade level**										
Junior high	*76*	27	18.9	49	14.8	26	18.7	45	13.8	0.152
Senior high	*398*	116	81.1	282	85.2	115	81.4	284	86.2	
**Sex**										
Male	*365*	110	76.9	255	77.0	108	76.1	251	76.2	0.969
Female	*109*	33	23.1	76	23.0	34	23.9	78	23.8	
**Father’s highest education level**										
Below junior high school	*136*	49	34.3	87	26.3	48	34.1	89	26.9	0.265
Senior or vocational high school	*227*	60	42.0	167	50.5	59	42.0	162	49.0	
Above college	*111*	34	23.8	77	23.3	34	24.0	80	24.1	
**Mother’s ethnicity**										
Han Chinese	*387*	117	81.8	270	81.6	117	82.8	273	82.7	0.550
Indigenous	*57*	19	13.3	38	11.5	18	12.5	34	10.4	
Foreigner	*30*	7	4.9	23	6.9	7	4.7	23	6.9	
**Parents’ employment status**										
Full-time	*428*	125	87.4	303	91.5	124	87.8	304	92.1	0.196
Part-time	*18*	5	3.5	13	3.9	5	3.6	13	3.9	
Unemployed	*28*	13	9.1	15	4.5	12	8.6	14	4.1	
**Family living arrangement**										
Parents or extended family	*302*	92	64.3	210	63.4	93	66.0	214	65.0	0.617
Single parent	*108*	30	21.0	78	23.6	28	20.1	77	23.5	
Grandparents	*29*	8	5.6	21	6.3	7	5.2	19	5.9	
Other relatives	*35*	13	9.1	22	6.6	12	8.7	19	5.7	

	Mean (SD)	Mean	SD	Mean	SD	Mean	SD	Mean	SD	*P* value
Change in total monthly combustible cigarette consumption from baseline to follow-up^a^	74.1(148.9)	72.1	144.0	78.6	160.3	77.1	163.3	70.7	143.7	0.705
CES-D Scale	*8.5(2.8)*	8.8	2.9	8.4	2.8	*8.8*	2.9	8.5	*2.8*	*0.170*
Peer Support Score	*13.4(2.5)*	13.2	2.6	13.6	2.4	*13.1*	2.6	13.6	2.5	*0.135*
Age	*17.9(1.3)*	17.8	1.3	18.0	1.3	*17.8*	1.3	18.1	1.3	*0.050*

Significant values are in italics.

CES-D Scale: Center for Epidemiologic Studies Depression Scale.

***p value < 0.001; **p value < 0.01; *p value < 0.05.

^a^Samples used for analysis are restricted to current smoker at both baseline and follow-up. Current smokers are referred to those who are combustible cigarette users.

### Association between use of e-cigarettes at baseline and smoking cessation in adolescent smokers

In Table 2, our findings indicated that for all current adolescent smokers, using e-cigarettes was not associated with smoking cessation as compared to current adolescent smokers who did not use e-cigarettes (aRR = 0.99; CI = 0.66, 1.50). Among junior high students who smoke, the use of e-cigarettes at baseline was not associated with smoking cessation as compared to junior high students who smoke but did not use e-cigarettes (aRR = 0.72; CI = 0.41, 1.28). Among high school students who smoke, the use of e-cigarettes at baseline was also not associated with smoking cessation as compared to high school students who were combustible cigarette users (aRR = 1.06; CI = 0.66, 1.72).

**Table 2:** Logistic regression model for adjusted risk ratios (aRR) of smoking cessation a stratified by age—weighted estimates (current smokers at baseline, n = 474)*.*

	All students			Junior high			Senior high		
	aRR	95% CI	P value	aRR	95% CI	P value	aRR	95% CI	P value
**Unadjusted model**									
Current use of E-cigarettes at baseline	1.00	0.69–1.47	0.98	0.79	0.42–1.48	0.46	1.05	0.67–1.64	0.84
**Adjusted model**^**a**^									
Current use of E-cigarettes at baseline	0.99	0.66–1.50	0.97	0.72	0.41–1.28	0.27	1.06	0.66–1.72	0.80

*aR*R, adjusted risk ratios; CES-D Scale, Center for Epidemiologic Studies Depression Scale.

***p value < 0.001; **p value < 0.01; *p value < 0.05.

^a^In this model, 1 is defined as successful smoking cessation and 0 is defined as still a current smoker. Therefore, a risk ratio of > 1 shows an increased probability of smoking cessation and a risk ratio of < 1 shows a decreased probability of smoking cessation. The multivariable logit regression model was adjusted for ever use of other tobacco products, depression (CES-D), peer support, father’s education, mother’s ethnicity, parents’ employment status, sex, age, and family living arrangement.

**Figure 2:**
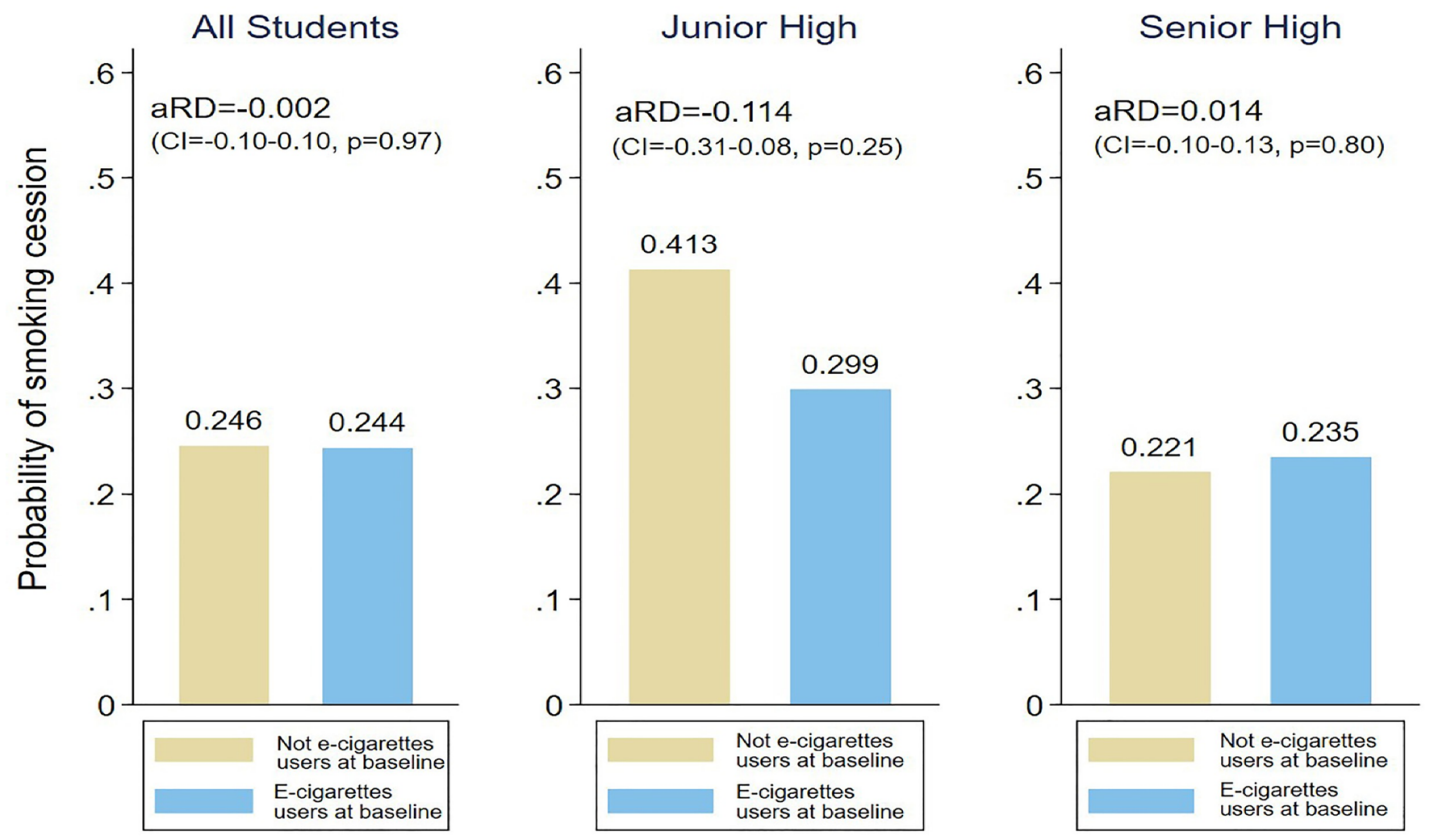
Logistic regression model for adjusted risk differences (aRD) of smoking cessation stratified by age—weighted estimates (Current smokers at baseline, *n* = 474)*. Note*: Models were adjusted by ever use of other tobacco products, depression (CES-D), peer support, father’s education, mother’s ethnicity, parents’ employment status, sex, age, and family living arrangement.

In Fig. 2, we used adjusted risk difference (aRD) to examine the effect that use of e-cigarettes has on the likelihood of adolescent smokers quitting smoking. The results of our research showed that for current adolescent smokers, using e-cigarettes was not associated with smoking cessation as compared to current adolescent smokers who did not use e-cigarettes (24.4% and 24.6%, respectively)(aRD = − 0.2%; CI − 0.10, 0.10; *p*=0.97). When we further stratified by age, among junior high students who smoke, the use of e-cigarettes was not associated with smoking cessation (aRD = 11.4%; CI 0.31–0.08, p = 0.25). Similarly, among high school students who smoke, the use of e-cigarettes was also not associated with smoking cessation (aRD = 1.4%; CI 0.10, 0.13; *p *= 0.80).

### Association between the change in cigarette consumption and the use of e-cigarettes

In Table 3, we further performed a regression model to investigate whether or not the use of e-cigarettes can aid in reducing combustible cigarette consumption. Among all students, e-cigarette use was found to have no association with change in combustible cigarette consumption (Coef. = 0.62; CI = − 36.85, 38.09). Among junior high school students, e-cigarette use was not associated with change in combustible cigarette consumption (coef. =  66.42; CI = − 2.76, 135.60). Similarly, among high school students, e-cigarette use was also not associated with change in cigarette consumption (coef. = 13.46; CI = − 51.49, 24.56).

**Table 3:** Multivariable regression model for change in total monthly combustible cigarette consumption from baseline to follow-up stratified by age—weighted estimates (current smokers at baseline and follow-up, *n* = 362)*.*

	All students		Junior high		High school	
	Coef.	95% CI	Coef.	95% CI	Coef.	95% CI
**Unadjusted model**						
Current use of E-cigarettes at baseline	6.30	− 30.95 to 43.55	38.36	− 39.83 to 116.55	− 0.51	− 42.09 to 41.08
**Adjusted model**						
Current use of E-cigarettes at baseline	0.62	− 36.85 to 38.09	66.42	− 2.76 to 135.60	− 13.46	− 51.49 to 24.56

Significant values are in italics.

CES-D Scale: Center for Epidemiologic Studies Depression Scale.

***p value < 0.001; **p value < 0.01; *p value < 0.05. The multivariable regression model was adjusted for ever use of other tobacco products, depression (CES-D), peer support, father’s education, mother’s ethnicity, parents’ employment status, sex, age, and family living arrangement.

## Discussion

Our present study aims to test two hypotheses: (1) whether the use of e-cigarettes at baseline can be helpful in combustible cigarette smoking cessation, and (2) how the use of e-cigarettes at baseline affects combustible cigarette consumption at follow-up among adolescent smokers. Our results suggested that the use of e-cigarettes at baseline was not associated with smoking cessation in adolescent combustible cigarette users. Moreover, the use of e-cigarettes at baseline was not associated with change in combustible cigarette consumption among adolescent combustible cigarette users.

Evidence from observational studies has been mixed regarding the association between e-cigarette use and combustible cigarette smoking cessation. In our current study, we hypothesized that the use of e-cigarettes at baseline cannot aid in quitting combustible cigarettes smoking. Some studies show that e-cigarettes may be helpful in reducing combustible tobacco consumption and increasing the likelihood of combustible cigarette smoking cessation^19,34^. However, other observational studies showed the opposite—e-cigarettes were not associated with increased combustible cigarette smoking cessation in both youth and adult populations^35,36^. The 2012 National Youth Tobacco Survey in Taiwan didn’t find any statistically significant association between e-cigarette use and intention to quit combustible cigarette smoking among current youth smokers^37^. The subsequent 2014 National Youth Tobacco Survey also concluded that, among current adolescent smokers, using flavored e-cigarettes was associated with a reduced likelihood of quitting combustible tobacco use in the next 12 months compared with non-e-cigarettes users^36^. Our study revealed similar results in that the use of e-cigarettes in younger adolescents, (i.e. junior high school students), was not associated with as statistically increased odds of quitting and was not associated with a statistically significant probability of decreasing combustible cigarettes consumption. Because the association between e-cigarette use and smoking cessation behavior remains unclear, more empirical research is warranted to help form evidence-based regulatory policy on e-cigarettes use for adolescents.

Among adolescents, there are still some behavioral differences exist between middle school students and high school students. Our findings in older adolescents, (i.e. high school students) suggest that e-cigarettes use may have some effect on combustible cigarette smoking cessation and reducing combustible cigarette consumption. However, this benefit must be balanced with the risk of younger adolescents, who are not current smokers and who were unlikely to try combustible cigarettes, experimenting with e-cigarettes and developing nicotine addictions. A recent nationwide study of youth drug use in the US, found that among several different ages of adolescents, only 5–10% of adolescents who had used e-cigarettes reported they used them for the purpose of reducing their combustible tobacco consumption. The majority stated that they used e-cigarettes because they wanted to see what they were like or liked the flavor^38^. Furthermore, epidemiological studies and population surveys also indicate that many e-cigarettes users with the initial intention of quitting ended up becoming dual users, especially in places where smoking is prohibited^39,40^. A web-based survey consisted of 4444 students from 8 colleges in North Carolina was completed in 2009 showed that e-cigarette use was not associated with intentions to quit smoking among a subsample of conventional cigarette smokers. Unlike older, more established combustible cigarette users, e-cigarette use by college students was not for the intention to quit smoking^41^.

Our current study not only attempts to answer the question of whether e-cigarette use is effective in smoking cessation, we also want to raise a health risk—e-cigarettes may perpetuate or initiate nicotine addiction. A study conducted by Truth Initiative and the US Centers for Disease Control and Prevention found that nearly all e-cigarettes sold by U.S. retailers contained nicotine, with the average nicotine concentration in e-cigarette products increasing from 2.10% to 4.34% between 2013 to 2018^42^. E-cigarette products containing no nicotine accounted for only 1% or less of the market share. The majority of youth e-cigarette users think they vaped only flavoring, not nicotine, according to the University of Michigan 2016 Monitoring the Future Study^42^. In Taiwan, nicotine containing e-cigarettes are illegal, but not well regulated and are widely available. According to a study conducted by the Taiwanese Food and Drug administration, out of a sample of 3062 e-cigarettes purchased from by retailers, about 80% contained nicotine^43^. The high nicotine concentrations found in e-cigarettes have their own health risks (i.e. lung disease, impaired brain development, future addiction to other drugs), and developing a nicotine addiction in early adolescence may increase the risk of consuming combustible cigarettes later in life.

Our present study takes a unique approach by comparing the effect of e-cigarettes on cessation among different age populations. A previous study from the United States found that e-cigarette use was associated with an increased likelihood of quitting combustible cigarette smoking among adult smokers attempting to quit combustible cigarette smoking^21^. We believe that social and cultural factors play a significant role in the effectiveness of using e-cigarettes in smoking cessation and that’ why our study obtains opposite results. Additionally, there may be important differences between adolescents and adults especially in terms of whether or not they have already developed nicotine dependence, and therefore whether e-cigarettes represent a quitting aid or a possible gateway drug^44^. It’s also possible that e-cigarettes are a useful tool among combustible cigarette users only if the users are also strongly motivated to quit^45^.

A limitation of our study is that despite TAALS being a nationally representative survey of Taiwanese adolescents, there were a low number of participants who smoked in our study population because the Taiwanese adolescent smoking rate is low at approximately 3.4%. As a result, out of our original cohort of 14,109 records, there were only 474 participants who smoked at baseline and therefore only 474 participants whose records could be analyzed to examine the effect of e-cigarette use on smoking cessation. Additionally, our study questionnaire lacks information regarding father’s smoking status, which is also an important factor affecting adolescents’ attitude towards smoking.

## Conclusions

This nationwide, representative study in Taiwan indicates there was no association between use of e-cigarettes and smoking cessation in adolescent combustible cigarette users. Furthermore, the use of e-cigarettes was also not associated with change in combustible cigarette consumption among adolescent combustible cigarette users. Meanwhile, e-cigarettes may hinder tobacco control efforts and therefore, e-cigarette regulations should pay special attention to the potential health effects of e-cigarette use among adolescents. It is essential for public health professionals and policymakers to understand the epidemiology of e-cigarette use and relevant correlates within each subgroup of age and smoking status to meet tobacco control policy goals.

## Data Availability

The data that support the findings of this study are available from the Taiwan Health Promotion Administration but are restricted for research use only. The data are not publicly available. Data are available from the authors upon reasonable request and with permission of the Taiwan Health Promotion Administration.

## References

[1] National Center for Chronic Disease Prevention and Health Promotion (US) Office on Smoking and Health. E-Cigarette Use Among Youth and Young Adults: A Report of the Surgeon General [Internet]. Atlanta (GA): Centers for Disease Control and Prevention (US); 2016 (Accessed on December 15th, 2021). 30869850

[2] National Center for Chronic Disease Prevention and Health Promotion (US) Office on Smoking and Health. E-Cigarette Use Among Youth and Young Adults: A Report of the Surgeon General [Internet]. Atlanta (GA): Centers for Disease Control and Prevention (US); 2016 (Assessed on December 20th, 2021). 30869850

[3] Gentzke AS, Jamal A (2020). Tobacco product use among middle and high school students, United States. MMWR Morb. Mortal. Wkly. Rep..

[4] Cullen KA, Gentzke AS, Sawdey MD, Chang JT, Anic GM, Wang TW, Creamer MR, Jamal A, Ambrose BK, King BA (2019). E-cigarette use among youth in the United States, 2019. JAMA.

[5] Wang X, Zhang X, Xu X, Gao Y (2018). Electronic cigarette use and smoking cessation behavior among adolescents in China. Addict. Behav..

[6] Wang TW, Gentzke AS, Creamer MR, Cullen KA, Holder-Hayes E, Sawdey MD, Anic GM, Portnoy DB, Hu S, Homa DM (2019). Tobacco product use and associated factors among middle and high school students—United States. Morbidity and mortality weekly report. Surveill. Summ. (Washington, DC).

[7] Palipudi KM, Mbulo L, Morton J, Mbulo L, Bunnell R, Blutcher-Nelson G, Kosen S, Tee GH, Abdalla AM, Mutawa KA (2016). Awareness and current use of electronic cigarettes in Indonesia, Malaysia, Qatar, and Greece: Findings From 2011–2013 global adult tobacco surveys. Nicot. Tob. Res. Off. J. Soc. Res. Nicot. Tob..

[8] Jiang N, Chen J, Wang MP, McGhee SM, Kwong AC, Lai VW, Lam TH (2016). Electronic cigarette awareness and use among adults in Hong Kong. Addict. Behav..

[9] Chen YL, Wu SC, Chen YT, Hsiao PC, Yu YH, Ting TT, Chen CY, Tu YK, Huang JH, Yang HJ (2019). E-cigarette use in a country with prevalent tobacco smoking: A population-based study in Taiwan. J. Epidemiol..

[10] Chen PC, Chang LC, Hsu C, Lee YC (2019). Dual use of E-cigarettes and traditional cigarettes among adolescents in Taiwan, 2014–2016. Nicot. Tob. Res. Off. J. Soc. Res. Nicot. Tob..

[11] Tsai J, Walton K, Coleman BN, Sharapova SR, Johnson SE, Kennedy SM, Caraballo RS (2018). Reasons for electronic cigarette use among middle and high school students—National Youth Tobacco Survey, United States, 2016. MMWR Morb. Mortal. Wkly. Rep..

[12] Rahman MA, Hann N, Wilson A, Worrall-Carter L (2014). Electronic cigarettes: Patterns of use, health effects, use in smoking cessation and regulatory issues. Tob. Induc. Dis..

[13] Polosa R, Rodu B, Caponnetto P, Maglia M, Raciti C (2013). A fresh look at tobacco harm reduction: The case for the electronic cigarette. Harm Reduct. J..

[14] National Academies of Sciences, E.; Medicine; Health; Medicine, D.; Board on Population, H.; Public Health, P.; Committee on the Review of the Health Effects of Electronic Nicotine Delivery, S. In *Public Health Consequences of E-Cigarettes*, Eaton, D. L., Kwan, L. Y., Stratton, K., Eds.; National Academies Press (US) Copyright 2018 by the National Academy of Sciences. All rights reserved, Washington (DC) (2018).

[15] Vardavas CI, Anagnostopoulos N, Kougias M, Evangelopoulou V, Connolly GN, Behrakis PK (2012). Short-term pulmonary effects of using an electronic cigarette: Impact on respiratory flow resistance, impedance, and exhaled nitric oxide. Chest.

[16] Evangelia Gkoulitou C (2020). The health effects of electronic cigarette use: An overview. Res Rev Insights.

[17] Bhatnagar A, Whitsel LP, Ribisl KM, Bullen C, Chaloupka F, Piano MR, Robertson RM, McAuley T, Goff D, Benowitz N (2014). Electronic cigarettes. Circulation.

[18] Chien YN, Gao W, Sanna M, Chen PL, Chen YH, Glantz S, Chiou HY (2019). Electronic cigarette use and smoking initiation in Taiwan: Evidence from the first prospective study in Asia. Int. J. Environ. Res. Public Health.

[19] Caponnetto P, Russo C, Bruno CM, Alamo A, Amaradio MD, Polosa R (2013). Electronic cigarette: A possible substitute for cigarette dependence. Monaldi Arch. Chest. Dis..

[20] Hajek P, Phillips-Waller A, Przulj D, Pesola F, Myers Smith K, Bisal N, Li J, Parrott S, Sasieni P, Dawkins L (2019). A randomized trial of E-cigarettes versus nicotine-replacement therapy. New Engl. J. Med..

[21] Benmarhnia T, Pierce JP, Leas E, White MM, Strong DR, Noble ML, Trinidad DR (2018). Can E-cigarettes and pharmaceutical aids increase smoking cessation and reduce cigarette consumption? Findings from a nationally representative cohort of American smokers. Am. J. Epidemiol..

[22] Bullen C, Howe C, Laugesen M, McRobbie H, Parag V, Williman J, Walker N (2013). Electronic cigarettes for smoking cessation: A randomised controlled trial. Lancet (London, England).

[23] Cobb C, Villanti A, Graham A, Pearson J, Glasser A, Rath J, Stanton C, Levy D, Abrams D, Niaura R (2015). A Markov model to estimate population-level patterns of cigarette and e-cigarette use. Tob. Regul. Sci..

[24] Levy DT, Borland R, Villanti AC, Niaura R, Yuan Z, Zhang Y, Meza R, Holford TR, Fong GT, Cummings KM (2017). The application of a decision-theoretic model to estimate the public health impact of vaporized nicotine product initiation in the United States. Nicot. Tob. Res. Off. J. Soc. Res. Nicot. Tob..

[25] Zhang YY, Bu FL, Dong F, Wang JH, Zhu SJ, Zhang XW, Robinson N, Liu JP (2021). The effect of e-cigarettes on smoking cessation and cigarette smoking initiation: An evidence-based rapid review and meta-analysis. Tob. Induc. Dis..

[26] Kalkhoran S, Glantz SA (2016). E-cigarettes and smoking cessation in real-world and clinical settings: A systematic review and meta-analysis. Lancet Respir. Med..

[27] Health Promotion Administrative, Ministry of Health and Welfare. Taiwan Global Youth Tobacco Survey (GYTS) (2022). Available online: www.hpa.gov.tw/Pages/Detail.aspx?nodeid=1725&pid=9931. Accessed on September 5th, 2022.

[28] Chien YN, Chen PL, Chen YH, Chang HJ, Yang SC, Chen YC, Chiou HY (2018). The Taiwan adolescent to adult longitudinal study (TAALS): Methodology and cohort description. Asia Pac. J. Public Health.

[29] Epstein M, Bailey JA, Kosterman R, Rhew IC, Furlong M, Oesterle S, McCabe SE (2021). E-cigarette use is associated with subsequent cigarette use among young adult non-smokers, over and above a range of antecedent risk factors: A propensity score analysis. Addiction (Abingdon, England).

[30] Lewinsohn PM, Seeley JR, Roberts RE, Allen NB (1997). Center for Epidemiologic Studies Depression Scale (CES-D) as a screening instrument for depression among community-residing older adults. Psychol. Aging.

[31] Radloff LS (1977). The CES-D Scale: A self-report depression scale for research in the general population. Appl. Psychol. Meas..

[32] Hamburger, M.E.; Basile, K.C.; Vivolo, A.M. Measuring bullying victimization, perpetration, and bystander experiences; a compendium of assessment tools (2011).

[33] Norton EC, Miller MM, Kleinman LC (2013). Computing adjusted risk ratios and risk differences in stata. Stand. Genomic Sci..

[34] Caponnetto P, Campagna D, Cibella F, Morjaria JB, Caruso M, Russo C, Polosa R (2013). EffiCiency and safety of an eLectronic cigAreTte (ECLAT) as tobacco cigarettes substitute: A prospective 12-month randomized control design study. PLoS ONE.

[35] Wang RJ, Bhadriraju S, Glantz SA (2021). E-cigarette use and adult cigarette smoking cessation: A meta-analysis. Am. J. Public Health.

[36] Dai H, Hao J (2016). Flavored electronic cigarette use and smoking among youth. Pediatrics.

[37] Park JY, Seo DC, Lin HC (2016). E-cigarette use and intention to initiate or quit smoking among US youths. Am. J. Public Health.

[38] Miech, R. A., Johnston, L. D., O'Malley, P. M., Bachman, J. G., & Schulenberg, J. E. *Monitoring the Future National Survey Results on DRUG use, 1975–2015: Volume I, Secondary School Students* (2016).

[39] Goniewicz ML, Zielinska-Danch W (2012). Electronic cigarette use among teenagers and young adults in Poland. Pediatrics.

[40] Kralikova E, Novak J, West O, Kmetova A, Hajek P (2013). Do e-cigarettes have the potential to compete with conventional cigarettes? A survey of conventional cigarette smokers' experiences with e-cigarettes. Chest.

[41] Sutfin EL, McCoy TP, Morrell HER, Hoeppner BB, Wolfson M (2013). Electronic cigarette use by college students. Drug Alcohol Depend..

[42] Romberg AR, Miller Lo EJ, Cuccia AF, Willett JG, Xiao H, Hair EC, Vallone DM, Marynak K, King BA (2019). Patterns of nicotine concentrations in electronic cigarettes sold in the United States, 2013–2018. Drug Alcohol Depend..

[43] Health Promotion Administrative, Ministry of Health and Welfare. Do Not Let the Electronic Cigarette Hurt You during the Summer Vacation (2017). Available online: https://www.hpa.gov.tw/Pages/Detail.aspx?nodeid=1137&pid=7654 (Accessed on December 20th, 2022).

[44] Carroll Chapman SL, Wu L-T (2014). E-cigarette prevalence and correlates of use among adolescents versus adults: A review and comparison. J. Psychiatr Res..

[45] Brandon KO, Simmons VN, Meltzer LR, Drobes DJ, Martínez Ú, Sutton SK, Palmer AM, Bullen CR, Harrell PT, Brandon TH (2019). Vaping characteristics and expectancies are associated with smoking cessation propensity among dual users of combustible and electronic cigarettes. Addiction (Abingdon, England).

